# Gain of 1q21 is an adverse prognostic factor for multiple myeloma patients treated by autologous stem cell transplantation: A multicenter study in China

**DOI:** 10.1002/cam4.3254

**Published:** 2020-09-02

**Authors:** Wen Gao, Yuan Jian, Juan Du, Xiaozhe Li, Huixing Zhou, Zhiyao Zhang, Guangzhong Yang, Guorong Wang, Ying Tian, Yanchen Li, Yin Wu, Weijun Fu, Juan Li, Wenming Chen

**Affiliations:** ^1^ Department of Hematology Myeloma Research Center of Beijing Beijing Chaoyang Hospital Capital Medical University Beijing China; ^2^ Department of Hematology The Myeloma & Lymphoma Center Changzheng Hospital The Second Military Medical University Shanghai China; ^3^ Department of Hematology The First Affiliated Hospital of Sun Yat‐Sen University Guangzhou China

**Keywords:** 1q21 gain, autologous stem cell transplantation, Multiple myeloma, prognosis

## Abstract

**Background:**

Autologous stem cell transplantation (ASCT) has been recommended as a standard approach for young multiple myeloma (MM) patients for decades, even in the era of novel agents. Gain of chromosome 1q21 is a common cytogenetic abnormality in MM, while its clinical prognostic value is still controversial.

**Methods:**

In this multicenter study, we retrospectively analyzed 1q21 gain in 446 newly diagnosed MM patients who received at least one ASCT from three large myeloma centers in China.

**Results:**

Of the all 446 patients, 1q21 gain was an adverse predictor of progression‐free survival (PFS) (34 vs 56 months, *P* = .005) and overall survival (OS) (69 vs 100 months, *P* = .002). Gain of 1q21 was more likely to coexist with t(4;14), t(14;16), and del(13q). Nevertheless, isolated 1q21 gain still exhibited unfavorable effects on PFS (35 vs 66 months, *P* = .045) and OS (61 vs 100 months, *P* = .026). The coexistence of 1q21 gain and high‐risk cytogenetics (HRCs) [del(17p), t(4;14),and/or t(14;16)] showed poor prognosis on both PFS and OS, while no additional adverse effect could be identified when compared with HRCs alone. Moreover, when coexisting with t(11;14), patients with 1q21 gain showed a comparable survival to those without 1q21 gain. For patients treated with novel induction regimens followed by ASCT, 1q21 gain also conferred an inferior prognosis. Multivariate analysis further confirmed 1q21 gain could independently predict shorter PFS and OS.

**Conclusion:**

In conclusion, 1q21 gain is an adverse prognostic factor for MM patients received ASCT.

## Graphical abstract

Gain of 1q21 is an independent adverse prognostic factor in MM patients receiving ASCT in the era of novel agents. Isolated 1q21 gain still showed adverse impact on patient's survival.

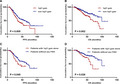

## 1 INTRODUCTION

Multiple myeloma (MM) is a clonal plasma cell malignancy characterized by heterogeneous cytogenetic abnormalities, resulting in a wide heterogeneity in survival outcomes.^1^, ^2^ Karyotypes of malignant plasma cells are typically complex, containing numerous numerical and structural defects, including chromosomal translocations, deletions, duplications, and genetic mutations.^3^ Among these, several cytogenetic abnormalities detected by interphase fluorescence in situ hybridization (FISH) has been recommended as a routine procedure for risk stratification and prognosis.^4^, ^5^ Autologous stem cell transplantation (ASCT) has been recommended as a standard approach as a part of upfront therapy for eligible newly diagnosed MM patients in the era of conventional chemotherapy,^6^ while still shows benefits on survival in the era of novel agents.^7^, ^8^, ^9^ Therefore, to figure out the biological role of the genetic basis in MM could contribute to individualized treatment and prediction of long‐term outcomes, especially in ASCT patients.

Previous studies have identified that some of the cytogenetic abnormalities could largely determine the clinical heterogeneity of MM. Based on general consensus, hyperdiploidy, t(11;14), and a normal karyotype are standard‐risk factors with a relatively favorable prognosis, while t(4;14), t(14;16), and del(17p) are high‐risk factors with an adverse prognosis.^5^, ^10^, ^11^, ^12^ However, within those cytogenetic abnormalities, the clinical prognostic value of 1q21 gain has been controversial. Some reports provided evidence that 1q21 gain was an independent prognostic factor related to shorter survival,^13^, ^14^, ^15^ whereas others failed to demonstrate its prognostic value.^16^ As the estimated survival of MM has improved substantially due to the prevalence of novel agents and ASCT, the prognostic value of 1q21 gain needs to be further clarified to provide a better understanding of the genetic basis and to predict long‐term outcomes of MM patients. In this context, we designed this multicenter retrospective study to assess the prognostic value of 1q21 gain in MM patients received ASCT.

## 2 MATERIALS AND METHODS

### 2.1 Patients

The data of 455 newly diagnosed symptomatic myeloma patients who received at least one ASCT after induction therapy with FISH detected at diagnosis from Beijing Chaoyang Hospital, Shanghai Changzheng Hospital, and the First Affiliated Hospital of Sun Yat‐sen University between March 2003 and Jan 2018 were collected. Nine patients were excluded for the absence of 1q results. Finally, a total of 446 patients were included in this study with 1q21 gain detected on a pretreatment bone marrow specimen. Patients who had amyloid‐related systemic syndrome or plasma cell leukemia at diagnosis were not enrolled in this study. The diagnostic criteria for symptomatic myeloma were defined by the International Myeloma Working Group (IMWG).^17^ Induction therapy regimens included bortezomib‐based regimens (n = 394), lenalidomide‐based regimens (with or without bortezomib, n = 7), and conventional regimens including TD, TAD, CTD, VAD, and CTAD (n = 45). All 446 patients received ASCT within 12 months of treatment initiation, of which eight patients received a second ASCT. Response to treatment was evaluated according to IMWG criteria,^18^ which was also retrospectively applied to evaluate patients before 2006. Follow‐up data were collected until January 2019. Median follow‐up duration was 36 (range 6‐120) months. Approval of this study was obtained from the Ethics Committee of Beijing Chaoyang Hospital. Written informed consents were obtained from all patients.

### 2.2 FISH analysis

FISH analysis was administrated in all patients before treatment. Of all the 446 patients, most (410 of 446 patients) bone marrow specimens were purified as CD138 positive plasma cells, while a few (36 of 446 patients) were not purified but directly analyzed as bone marrow mononuclear cells. These specimens were analyzed to detect the following cytogenetic aberrations: del(17p), t(4;14), t(11;14), t(14;16), del(13q), and 1q21 gain. A total of 200 interphase nuclei were analyzed. The cutoff values were as the following: 10% for t(4;14), t(11;14), and t(14;16), 20% for del(17p), del(13q), and 1q21 gains.^19^

### 2.3 Statistical analysis

The categorical clinical characteristics and cytogenetics were summarized as percentages, and continuous clinical characteristics were described as median and range. The chi‐squared test or two‐sided Fisher exact test was employed to compare categorical clinical characteristics and cytogenetics between the groups. Wilcoxon rank sum tests were employed to compare continuous clinical characteristics between the groups. Progression‐free survival (PFS) was defined as the duration from the initiation of chemotherapy to the first evidence of disease progression or death from any cause. Overall survival (OS) was defined as the duration from the initiation of therapy to death from any cause.^20^ The Kaplan‐Meier method was employed to plot the survival curves, with the log‐rank test to assess the differences. Cox proportional hazard regression analysis was employed to evaluate the prognostic value of the factors. SPSS version 24.0 (SPSS, Inc) was used for all statistical analyses. Statistical significance was reached if the *P*‐value was less than 0.05.

## 3 RESULTS

### 3.1 Clinical characteristics of 1q21 gain

Gain of 1q21 was detected in 39.7% (177/446) of newly diagnosed MM patients. The correlation between 1q21 gain and a variety of clinical characteristics was investigated in all the 446 patients (Table 1). Among all these factors, hemoglobin, age, and M component were found to be associated with 1q21 gain. Patients with 1q21 gain tended to have lower hemoglobin concentration than those without 1q21 gain (94 vs 102 g/L, *P* = .017). The median age of patients with 1q21 gain was slightly higher than those without 1q21 gain (54 vs 52 years old, *P* = .017). The proportion of M component was also different between the two groups of patients. Higher proportion of IgA (26% vs 17.8%) and lower proportion of nonsecretory M component (0.6% vs 4.8%) were observed in patients with 1q21 gain than those without 1q21 gain (*P* = .014). Statistical differences were not reached between 1q21 gain and non‐1q21 gain groups in other clinical baselines such as gender, DS stage, ISS stage, calcium, lactate dehydrogenase (LDH), serum creatinine, albumin, and β2‐microglobulin (*P* > .05), as well as in the aspects of induction, conditioning, and maintenance regimens (Table 1).

**TABLE 1:** Correlation between 1q21 gain and clinical characteristics

n = 446	Patients with 1q21 gain (n = 177)	Patients without 1q21 gain (n = 269)	*P* value
Gender			.768
Male	108/177 (61.0)	160/269 (59.5)	
Female	69/177 (39.0)	109/269 (40.5)	
Age (years)	54 (33‐68)	52 (23‐69)	.017*
DS stage			.142
Ⅰ	7/176 (4.0)	5/268 (1.9)	
Ⅱ	26/176 (14.8)	28/268 (10.4)	
Ⅲ	143/176 (81.2)	235/268 (87.7)	
ISS stage			.326
Ⅰ	44/177 (24.9)	81/269 (30.1)	
Ⅱ	62/177 (35.0)	97/269 (36.1)	
Ⅲ	71/177 (40.1)	91/269 (33.8)	
M component			.014*
IgG	90/177 (50.8)	144/269 (53.5)	
IgA	46/177 (26.0)	48/269 (17.8)	
IgD	10/177 (5.6)	8/269 (3.0)	
Light chain	30/177 (16.9)	56/269 (20.8)	
Nonsecretory	1/177 (0.6)	13/269 (4.8)	
Hemoglobin (g/L)	94 (44‐146)	102 (48‐159)	.017*
Calcium (mmol/L)	2.39 (1.65‐4.29)	2.37 (1.88‐4.11)	.612
Lactate dehydrogenase (U/L)	153 (67‐732)	164 (72‐704)	.190
Serum creatinine (μmol/L)	75.8 (30.0‐881.0)	78.1 (31.0‐777.0)	.276
Albumin (g/L)	34.6 (17.0‐55.0)	36.0 (17.4‐51.0)	.069
β2‐microglobulin (mg/L)	3.40 (0.63‐28.66)	3.22 (0.55‐46.60)	.768
Induction			.631
Bortezomib‐ or lenalidimide‐based	161/177 (91.0)	240/269 (89.2)	
Conventional	16/177 (9.0)	29/269 (10.8)	
Conditioning			.503
Melphalan‐based	92/173 (53.2)	149/264 (56.4)	
Busulfan‐based	81/173 (46.8)	115/264 (43.6)	
Maintenance			.548
Bortezomib‐ or lenalidimide‐based	42/177 (23.7)	54/269 (20.1)	
Conventional	115/177 (65.0)	188/269 (69.9)	
No	20/177 (11.3)	27/269 (10.0)	

Note

Data are presented as n (%) or median (range)

* Means *P* < .05

### 3.2 Overlap of 1q21 gain and other cytogenetic abnormalities

In total, 446 patients were analyzed for 1q21 gain, del(17p), t(4;14) and t(11;14), 440 of them were analyzed for t(14;16), and 297 of them were analyzed for del(13q) additionally. The correlation of 1q21 gain and other cytogenetic abnormalities was analyzed. Results revealed that 1q21 gain was more likely to coexist with other cytogenetic abnormalities. The incidence of other cytogenetic abnormalities, including t(4;14), t(14;16), t(11;14), del(17p), and del(13q), was 71.8% in 1q21 gain cases and 48.0% in non‐1q21 gain cases (*P* < .001). Further analysis revealed that the following three aberrations were more frequent in 1q21 gain cases than that in non‐1q21 gain cases, which were t(4;14) (26.0% vs 14.1%, *P* = .002), t(14;16) (5.6% vs 1.5%, *P* = .024), and del(13q) (57.4% vs 32.6%, *P* < .001), while del(17p) and t(11;14) exhibited no significant correlation with 1q21 gain (*P* > .05) (Table 2).

**TABLE 2:** Correlation of 1q21 gain with other cytogenetic abnormalities in MM

	Patients with 1q21 gain	Patients without 1q21 gain	*P*‐value
del(17p)	28/177 (15.8)	39/269 (14.5)	.787
t(4;14)	46/177 (26.0)	38/269 (14.1)	.002**
t(14;16)	10/177 (5.6)	4/263 (1.5)	.024*
t(11;14)	26/177 (14.7)	38/269 (14.1)	.891
del(13q)	70/122 (57.4)	57/175 (32.6)	<.001***
Total	127/177 (71.8)	129/269 (48.0)	<.001***

Note

Data are presented as n (%).

* Means *P* < .05,

** Means *P* < .01,

*** Means *P* < .001

### 3.3 Gain of 1q21 and response rate

Response to therapy was evaluated in patients before ASCT and 3 months after ASCT (Table 3). The response rate was similar in patients with or without 1q21 gain both before (*P* = .153) and after ASCT (*P* = .166). Moreover, although ASCT improved CR rates in both groups, patients without 1q21 gain had higher CR rate than patients with 1q21 gain after ASCT (49.8% vs 39.5%, *P* = .044), whereas the two groups did not show statistical differences before ASCT (35.7% vs 28.2%, *P* = .099), which indicated that patients with 1q21 gain were less likely to get deeper responses after ASCT than those without 1q21 gain.

**TABLE 3:** Response rate before and after ASCT according to 1q21 gain

Response	Before ASCT		Three months after ASCT	
	With 1q21 gain (n = 177)	Without 1q21 gain (n = 269)	With 1q21 gain (n = 177)	Without 1q21 gain (n = 269)
sCR	15 (8.5)	20 (7.4)	21 (11.9)	31 (11.5)
CR	35 (19.8)	76 (28.3)	49 (27.7)	103 (38.3)
VGPR	74 (41.8)	104 (38.7)	59 (33.3)	70 (26.0)
PR	41 (23.2)	53 (19.7)	23 (13.0)	35 (13.0)
SD/PD	11 (6.2)	14 (5.2)	10 (5.6)	12 (4.5)
Unknown	1 (0.6)	2 (0.7)	15 (8.5)	18 (6.7)

Note

Data are presented as n (%).

Abbreviations: ASCT, autologous stem cell transplantation; CR, complete response; PD, progressive disease; PR, partial response; sCR, stringent complete response; SD, stable disease; VGPR, very good partial response.

### 3.4 Survival analysis

**FIGURE 1:**
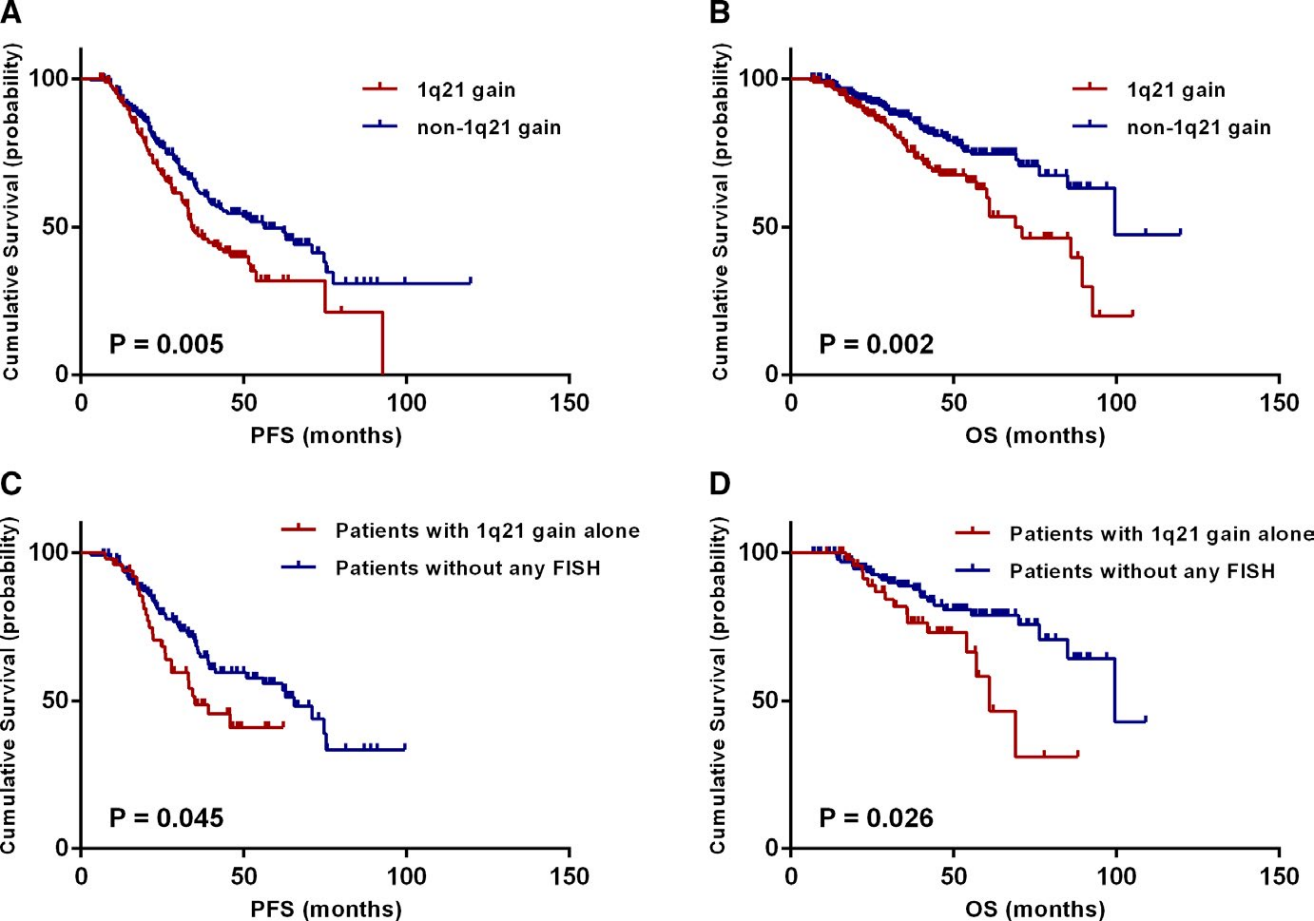
Effects of 1q21 gain on survival. A, B, PFS and OS in relation to 1q21 gain in all patients. C, D, PFS and OS in relation to isolated 1q21 gain in patients without other detectable FISH abnormalities [t(4;14), t(14;16), t(11;14), del(17p), and del(13q)]

Survival analysis was performed in order to assess the impact of 1q21 gain on PFS and OS in MM patients. The difference in survival between patients with and without 1q21 gain was first analyzed. Results revealed that PFS and OS of patients with 1q21 gain were shorter than those without 1q21 gain (median PFS: 34 vs 56 months, *P* = .005; median OS: 69 vs 100 months, *P* = .002) (Figure 1A,B). Taking into consideration that 1q21 gain was more likely to coexist with other cytogenetic abnormalities, especially the high‐risk cytogenetics, the comparisons were made between isolated 1q21 gain patients (n = 50) and FISH‐negative patients (n = 140). Data revealed that isolated 1q21 gain was also an adverse prognostic factor of PFS and OS (median PFS: 35 vs 66 months, *P* = .045; median OS: 61 vs 100 months, *P* = .026) (Figure 1C,D). Clinical characteristics between isolated and coexisted 1q21 gain were compared in Table S1.

### 3.5 Prognostic value of 1q21 gain at different clone sizes and copy numbers

**FIGURE 2:**
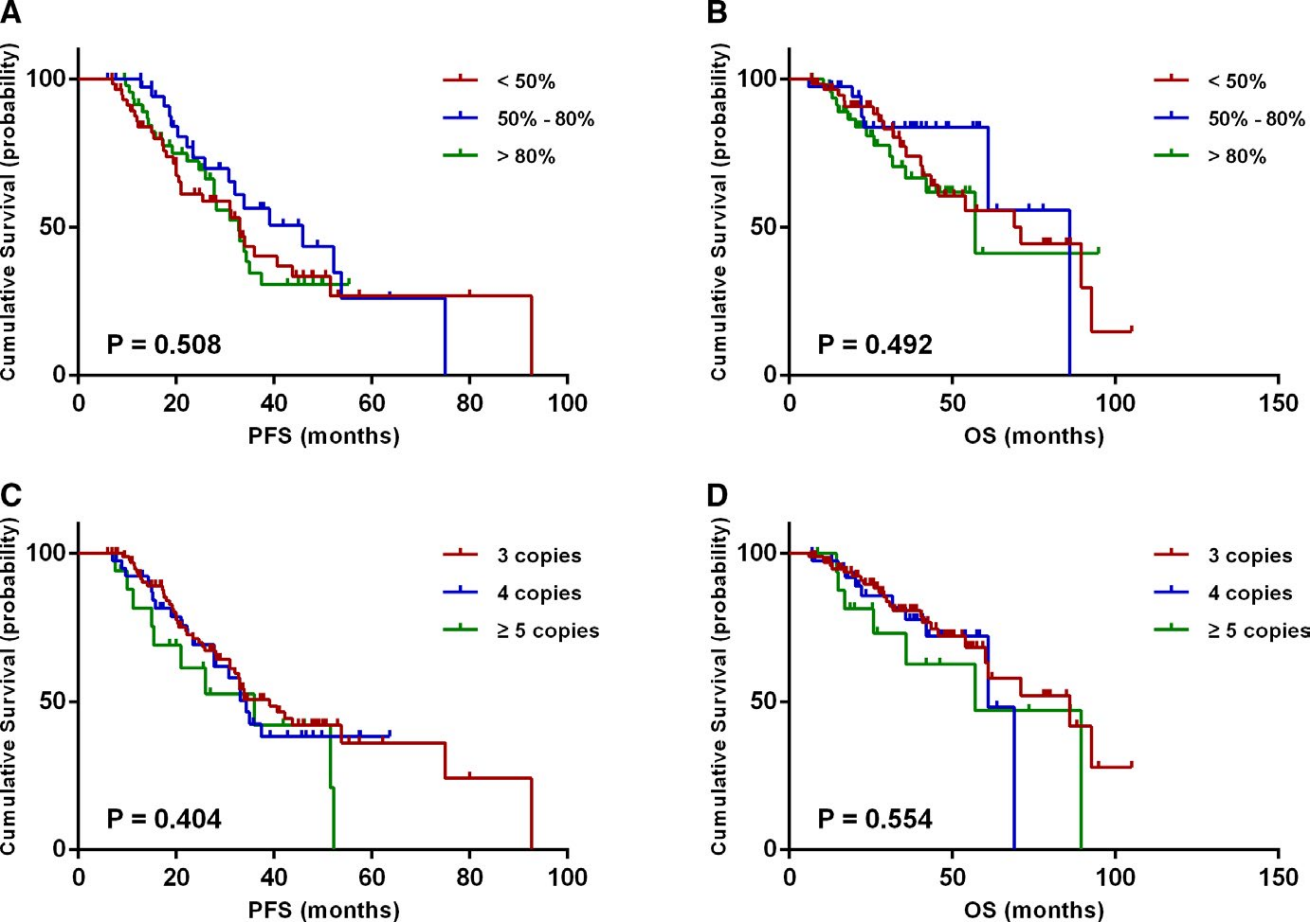
Effects of 1q21 gain clone sizes and copy numbers on survival. A, B, PFS and OS in relation to different 1q21 gain clone sizes in patients with 1q21 gain. C, D, PFS and OS in relation to different 1q21 copy numbers

The impacts of 1q21 gain on survival based on different clone sizes and copy numbers were analyzed. The median percentage of plasma cells harboring 1q21 gain among patients with 1q21 gain was 64.0% (range 20% to 100%). Patients with 1q21 gain were divided into three groups according to the percentage of plasma cells involved: <50%, 50%‐80%, and >80%. The median PFS times of the three groups were 33, 46, and 33 months, respectively. While the median OS times were 71, 86, and 57 months, respectively. No statistical significance was observed between different clone sizes in 1q21 gain positive patients in the aspect of both PFS (*P* = .508) and OS (*P* = .492) (Figure 2A,B).

The PFS and OS time of patients carried different 1q21 copy numbers on survival were further analyzed. With the results, patients carried 3, 4, or at least 5 copies of 1q21 gain and had median PFS times of 39, 34, and 36 months, respectively (*P* = .404), whereas the median OS times were 86, 61, and 57 months, respectively (*P* = .554) (Figure 2C,D). No statistical significance was shown between these differences.

### 3.6 Prognostic value of 1q21 gain coexisting with other cytogenetics

**FIGURE 3:**
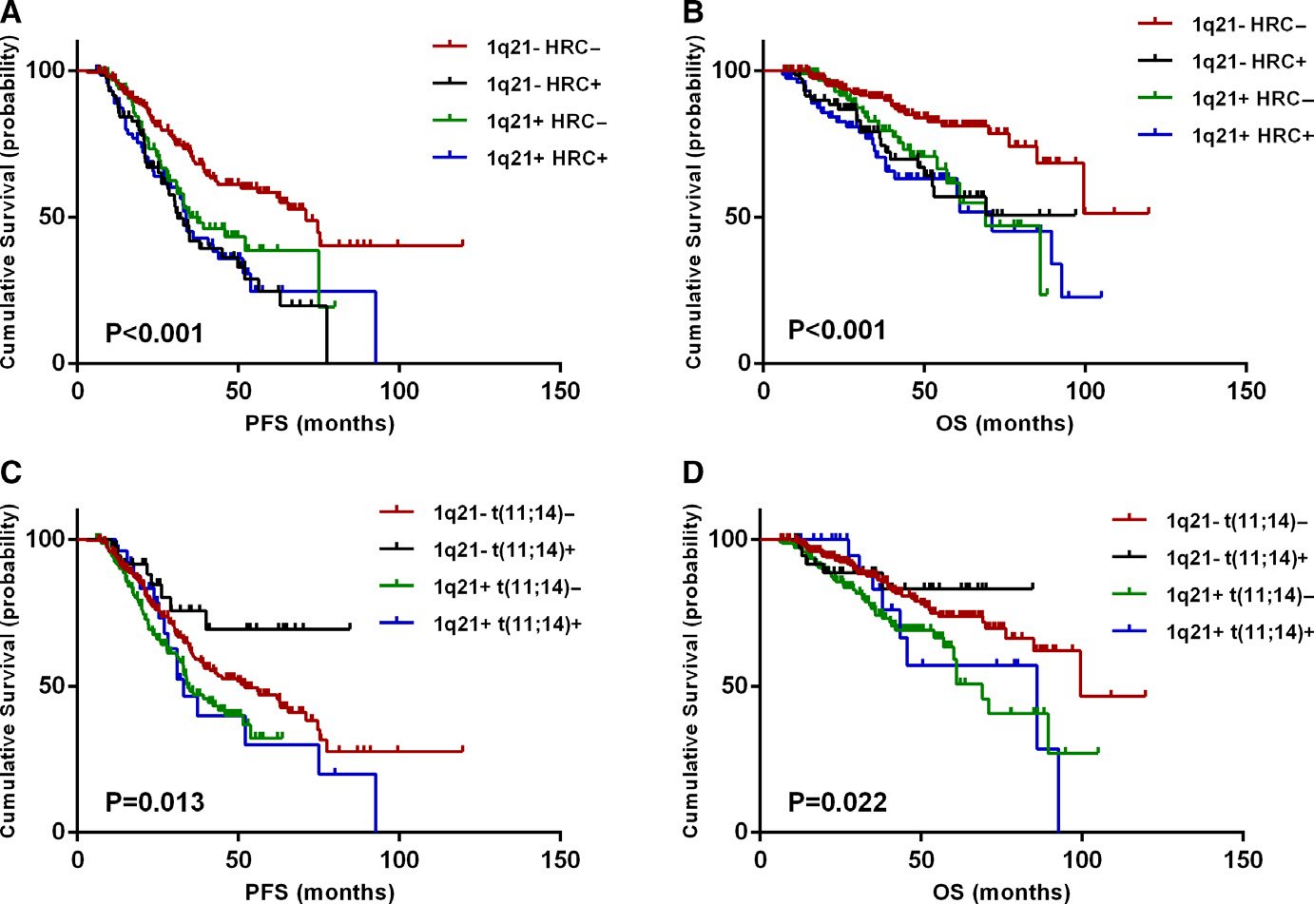
Effects of combinations of 1q21 gain and other cytogenetics on survival. A, B, PFS and OS in relation to coexistence of 1q21 gain and high‐risk cytogenetic abnormalities. C, D, PFS and OS in relation to coexistence of 1q21 gain and t(11;14) in patients without high‐risk cytogenetics [t(4;14), t(14;16) and del(17p)]

The combined effects of 1q21 gain and other cytogenetic abnormalities on patient outcomes were further analyzed. As to the routine risk stratification mentioned above,^4^, ^5^ del(17p), t(4;14), and t(14;16) were considered as high‐risk cytogenetics (HRCs) in the following analysis. Patients were divided into four groups according to 1q21 gain and HRCs. Results showed that PFS and OS among the four groups had significant differences (*P* < .001). Strikingly, patients without 1q21 gain without HRC (1q21‐HRC‐) showed longer PFS and OS time (71 months and not reached) than the other three groups (Figure 3A,B). No significant difference was found between 1q21 + HRC+, 1q21 + HRC−, and 1q21−HRC + groups.

Furthermore, the combined effects of 1q21 gain and t(11;14) were analyzed. Patients were also divided into four groups according to 1q21 gain and t(11;14). Among the four groups, patients without 1q21 gain with t(11;14) [1q21−t(11;14)+] had longer PFS and OS time than the other three groups (both not reached). Patients with both of above [1q21+t(11;14)+] had shorter PFS and OS than those carried neither [1q21−t(11;14)−] (median PFS: 33 vs 52 months, *P* = .268; median OS: 86 vs 100 months, *P* = .168); however, the difference did not show statistical significance. These results indicated that the coexistence of 1q21 gain and t(11;14) lost the superiority of t(11;14) in survival, but not show worse prognosis (Figure 3C,D).

### 3.7 Prognostic value of 1q21 gain in novel agents

**FIGURE 4:**
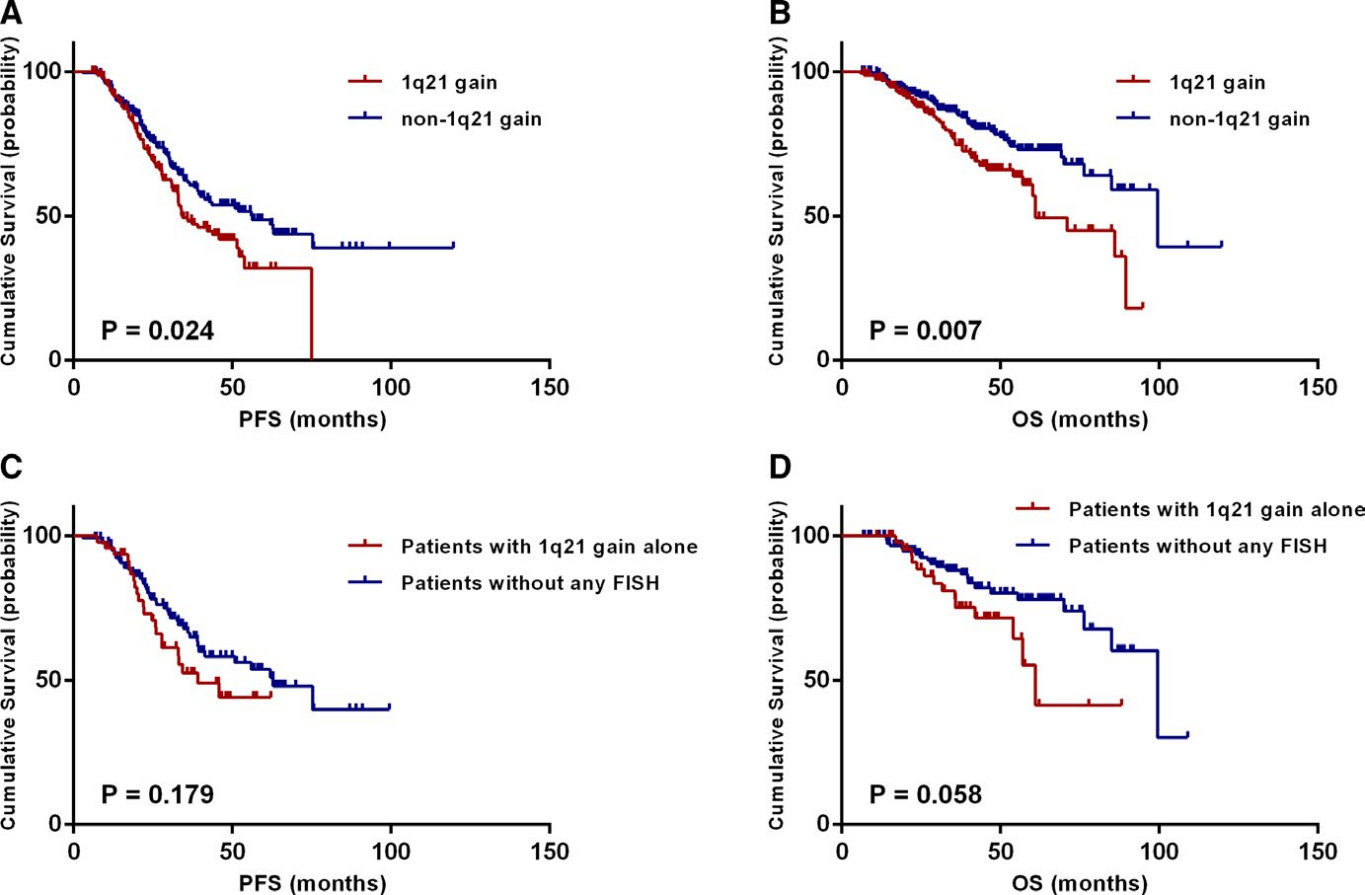
Effects of 1q21 gain on survival in novel agents (bortezomib or lenalidomide) based treatments. A, B, PFS and OS in relation to 1q21 gain in all novel agents treated patients. C, D, PFS and OS in relation to isolated 1q21 gain in novel agents treated patients without other detectable FISH abnormalities [t(4;14), t(14;16), t(11;14), del(17p), and del(13q)]

The effects of 1q21 gain in patients treated with novel agents (bortezomib or lenalidomide) were subsequently analyzed. In the all 401 patients treated with novel agents, those carried 1q21 gain had shorter PFS and OS times than non‐1q21 gain patients (median PFS: 34 vs 56 months, *P* = .024; median OS: 61 vs 100 months, *P* = .007) (Figure 4A,B). As all of our patients had received ASCT, these results revealed that patients without 1q21 gain derived a better PFS and OS benefit with bortezomib‐ or lenalidomide‐based induction therapies followed by ASCT relative to those with 1q21 gain. Moreover, survival differences between novel agents treated isolated 1q21 gain patients (n = 47) and FISH negative patients (n = 124) were further analyzed. Although the results did not show statistical differences, patients with isolated 1q21 gain had a tendency of shorter PFS and OS than those without any FISH abnormalities (median PFS: 39 vs 63 months, *P* = .179; median OS: 61 vs 100 months, *P* = .058) (Figure 4C,D).

### 3.8 Multivariate analysis

Univariate and multivariate analyses of PFS and OS were performed on cytogenetics and other clinical parameters. In univariate analyses, ISS stage Ⅲ, LDH ≥ 250 U/L, t(4;14), del(13q), and 1q21 gain were associated with shorter PFS; except for t(4;14), the above variables were also associated with shorter OS (Table 4). Then multivariate analysis containing the five parameters associated with survival in the univariate analyses was performed subsequently. Of these, 1q21 gain was statistically independent indicator of both PFS (HR 1.378, 95% CI: 1.003‐1.894, *P* = .048) and OS (HR 1.721, 95% CI: 1.121‐2.643, *P* = .013). In addition, LDH ≥ 250 U/L, del(17p), and t(4;14) were also independent indicators for PFS, with hazard ratios of 1.914 (95% CI: 1.296‐2.825, *P* = .001), 2.028 (95% CI: 1.392‐2.954, *P* < .001), and 1.531 (95% CI: 1.061‐2.209, *P* = .023), respectively; while LDH ≥ 250 U/L and del(17p) were also independent indicators for OS, with hazard ratios of 2.340 (95% CI: 1.391‐3.935, *P* = .001) and 2.674 (95% CI: 1.679‐4.261, *P* < .001), respectively (Table 5).

**TABLE 4:** Univariate analysis of variables associated with patient outcomes

Parameter	PFS			OS		
	HR	95% CI	*P* value	HR	95% CI	*P* value
Age (y) ≥50 vs <50	1.065	0.790‐1.434	.680	1.196	0.788‐1.816	.400
ISS stage III vs I‐II	1.390	1.041‐1.854	.025*	1.806	1.219‐2.678	.003**
Calcium (μmol/L) ≥ 2.6 vs < 2.6	1.301	0.907‐1.866	.153	1.131	0.677‐1.888	.638
Serum creatinine (μmol/L) ≥130 vs <130	1.180	0.823‐1.689	.368	1.551	0.974‐2.468	.064
Hemoglobin (g/L) <100 vs ≥100	1.158	0.864‐1.552	.327	1.183	0.788‐1.776	.418
Lactate dehydrogenase (U/L) ≥ 250 vs <250	2.130	1.454‐3.119	<.001***	2.748	1.656‐4.559	<.001***
del (17p) Positive vs negative	1.843	1.328‐2.559	<.001***	2.474	1.633‐3.747	<.001***
t(4;14) Positive vs negative	1.688	1.198‐2.380	.003**	1.368	0.852‐2.197	.195
t(11;14) Positive vs negative	0.765	0.494‐1.184	.230	0.960	0.535‐1.721	.891
Gain of 1q21 Positive vs negative	1.515	1.135‐2.023	.005**	1.837	1.239‐2.724	.002**
t(14;16) Positive vs negative	1.268	0.595‐2.700	.539	1.971	0.799‐4.863	.141

Abbreviations: CI, confidence interval; HR, hazard ratio.

* Means *P* < .05,

** Means *P* < .01,

*** Means *P* < .001

**TABLE 5:** Multivariate analysis of variables associated with patient outcomes

Parameter	PFS			OS		
	HR	95% CI	*P* value	HR	95% CI	*P* value
ISS stage III vs. I‐II	1.218	0.883‐1.679	.230	1.487	0.961‐2.299	0.075
Lactate dehydrogenase (U/L) ≥ 250 vs. < 250	1.914	1.296‐2.825	.001**	2.340	1.391‐3.935	0.001**
del (17p) Positive vs negative	2.028	1.392‐2.954	<.001***	2.674	1.679‐4.261	<0.001***
t(4;14) Positive vs negative	1.531	1.061‐2.209	.023*	—	—	—
Gain of 1q21 Positive vs negative	1.378	1.003‐1.894	.048*	1.721	1.121‐2.643	.013**

Abbreviations: CI, confidence interval;HR: hazard ratio.

* Means *P* < .05,

** Means *P* < .01,

*** Means *P* < .001

## 4 DISCUSSION

As ASCT has been a standard approach in MM for eligible patients, the risk stratification needs to be refined in ASCT patients. FISH analysis has been a routine detection in newly diagnosed MM patients, whereas the prognostic role of 1q21 gain has been controversial. Therefore, we obtained credible data from three myeloma centers in China about the 1q21 gain detected by interphase FISH and survival of Chinese MM patients. This study is the largest cohort from China to specify the clinical characteristics and outcomes of MM patients with 1q21 gain in the era of novel agents in combination with ASCT. With our results, 1q21 gain is an independent adverse indicator for both PFS and OS. Although 1q21 gain tends to be accompanied by other cytogenetic abnormalities, isolated 1q21 gain also exhibits an adverse prognostic effect on survival in MM patients received ASCT. The clone sizes and copy numbers of 1q21 gain do not show differences in patient outcomes. The coexistence of 1q21 gain and high‐risk cytogenetics would not confer a worse prognosis than HRCs alone, whereas the coexistence of 1q21 gain and t(11;14) lost the superiority of t(11;14).

Previous studies have found that 1q21 gain was one of the most frequent chromosomal aberrations in MM, with the occurrence rate of about 30% to 50%.^14^, ^21^, ^22^, ^23^ In the present study, 1q21 gain could be identified in 39.7% of all 446 patients, which is consistent with previously published studies. Thus, the biological characteristics and prognostic effect of 1q21 gain need to be investigated. Actually, 1q21 gain has been classified into the standard‐risk category by IMWG consensus published in 2014, while low risk must meet the criteria of absence of 1q21 gain.^4^ Although 1q21 gain was not specially mentioned and was considered as standard‐risk in 2013 Mayo mSMART consensus,^5^ revised Mayo mSMART 3.0 published in 2018 ASH meeting classified 1q21 gain into the high‐risk group. However, as it was mentioned above, the prognostic value of 1q21 gain is controversial. One possible explanation could be attributed to different treatment strategies. In the era of conventional chemotherapy, some studies demonstrated that 1q21 gain could be regarded as an independent prognostic indicator for PFS and OS in patients receiving conventional chemotherapy followed by ASCT.^22^, ^24^ Another research published by Grzasko et al^25^ found that 1q21 gain alone and with additional cytogenetic abnormalities both exhibited adverse impact on PFS and OS in MM. Nevertheless, another study published by Fonseca found that 1q21 gain lost its independent prognostic value in multivariate analysis,^16^ which indicated that the adverse impact of 1q21 gain might be contributed to other high‐risk factors. As to the era of novel agents, although the response rate and survival of MM have been largely improved, most studies supported the conclusion that 1q21 gain was an adverse indicator in patients treated with novel agents and ASCT,^26^, ^27^ while some demonstrated that 1q21 gain lost its prognostic value in bortezomib‐treated patients.^28^ A study of Asian patients analyzed the role of 1q21 gain in different treatment strategies, and found that the presence of 1q21 gain deterred PFS to bortezomib and lenalidomide, while ASCT was also less effective in patients with 1q21 gain.^29^ A recently published multicenter study from China also supported that 1q21 gain is an independent adverse prognostic indicator for PFS,^30^ despite of novel agents or ASCT, which was in agreement with our previous studies.^15^, ^31^ According to our results, 1q21 gain is independently associated with lower post‐ASCT CR rates and shorter PFS and OS in ASCT patients. In patients under bortezomib‐ or lenalidomide‐based chemotherapy and ASCT, 1q21 gain still conferred a significantly inferior prognosis than those without 1q21 gain. Novel therapeutic approaches or clinical trials might be needed to further improve the outcome of these patients.

Besides different treatment strategies, another important explanation of the inconsistency in the outcome of 1q21 gain is the coexistence of other cytogenetics. According to our data, patients with 1q21 gain had a higher incidence of del(17p), t(4;14) and del(13q) than those without 1q21 gain, which supports the unfavorable biological characteristics of 1q21 gain from another aspect. Some previous studies also reported the association between 1q21 gain and other unfavorable cytogenetics.^21^, ^22^ Importantly, for those who have 1q21 gain as the only detectable FISH aberration in our panel, 1q21 gain also related to shorter PFS and OS, indicating that 1q21 gain could confer inferior outcomes as an isolated chromosomal abnormality independently of HRCs. Moreover, when analyzing the combined effect of 1q21 gain and HRCs, we found that patients carried neither of the above had the most favorable outcomes. However, we could not identify additional adverse PFS and OS when 1q21 gain coexisted with other high‐risk cytogenetics, which is corresponding with our previous study and some other studies.^15^, ^30^, ^32^ On the other side, when coexisting with t(11;14), which is supposed to be a standard‐risk chromosomal aberration,^4^, ^5^ patients with 1q21 gain exhibit comparable survival to those without 1q21 gain, indicating that t(11;14) could at least partially overcome the adverse prognostic effect of 1q21 gain.

The prognostic value of 1q21 copy numbers has been analyzed. A meta‐analysis of 1905 newly diagnosed MM patients demonstrated that ≥ 4 copies of 1q21 was related to shorter PFS and OS, whereas 3 copies of 1q21 did not show prognostic significance.^33^ However, another study from China did not exhibit any difference in patient outcomes between different copy numbers of 1q21.^30^ Similarly, this study failed to demonstrate the prognostic significance of different clone sizes and copy numbers of 1q21 gain. Another research^32^ recently defined a high‐risk subgroup as “Double‐Hit,” which demonstrated patients with 1q21 gain (≥4 copies) on the background of ISS Ⅲ had an extremely poor outcome despite modern therapies, with a median PFS of only 15.4 months and OS of 20.7 months. In this study, 25 patients meeting the criteria of “Double‐Hit.” The median PFS and OS of our Double‐Hit patients were 35 months and 57 months, respectively. One of the possible explanations of their better survival is that all of the patients received ASCT, and 21 of 25 patients received novel agents based therapies, which might have improved the survival outcomes.

Another possible explanation of the heterogeneity of 1q21 gain was its clinical characteristics. As to our results, patients with 1q21 gain had a high incidence of IgA and a low incidence of nonsecretory subtypes. The correlation between different M subtypes and 1q21 gain was rarely reported,^34^ possibly due to its biological characteristics. Besides, the median age of patients with 1q21 gain was slightly older than those without 1q21 gain. Considering the fact that patients in our cohort all received ASCT, which means most of them were younger than 65 years old, the difference of median age may not clinically significant as the selection bias.

As a retrospective study, there are some limitations that should be considered. One main limitation is that only a small proportion of patients (137/446, 30.7%) were evaluated for chromosomal G banded karyotypes at diagnosis. So it is difficult to tell whether 1q gain occurs as an additional increase in copy number or is amplified more than the background chromosomal gains. Another limitation is the heterogeneity in terms of both induction and conditioning regimens. Further follow‐up and larger prospective studies are needed to verify the results.

In conclusion, the current study demonstrates that 1q21 gain is an independent adverse prognostic factor for MM patients with the use of novel agents–based induction regimens consolidated with ASCT, associating with reduced PFS and OS. FISH analysis of 1q21 gain should be used in routine assessment in MM patients at diagnosis to make a precise prognosis.

## AUTHOR CONTRIBUTIONS

WG and WC designed this study. GY, GW, YT, YL, and YW provided study patients and collected data. YJ, JD, XL, HZ, and ZZ performed data analysis. WG and YJ wrote the manuscript. WF, JL, and WC supervised the study.

## CONFLICT OF INTEREST

All authors declare that they have no conflict of interest.

## Supporting information

Table S1

## Data Availability

Research data are not shared.
