# Supplementary material for: Gain of 1q21 is an adverse prognostic factor for multiple myeloma patients treated by autologous stem cell transplantation: A multicenter study in China
Source: Cancer Med. 2020 Sep 2;9(21):7819–29. doi: 10.1002/cam4.3254 (PMC7643680; doi:10.1002/cam4.3254)
Supplement: Supplementary file 1 — Table S1 [file CAM4-9-7819-s001.docx]

**Supplementary Table 1**. Clinical characteristics between 1q21 gain alone and coexisting with other cytogenetic abnormalities

| n=177 |  |  |  |
| --- | --- | --- | --- |
|  | Patients with isolated 1q21 gain  (n=50) | Patients with 1q21 gain coexisting with other cytogenetics (n=127) | *P* value |
| Gender |  |  | 0.866 |
| Male | 30/50 (60.0) | 78/127 (61.4) |  |
| Female | 20/50 (40.0) | 49/127 (38.6) |  |
| Age (years) | 56 (33-66) | 53 (35-68) | 0.024* |
| DS stage |  |  | 0.959 |
| Ⅰ | 2/50 (4.0) | 5/126 (4.0) |  |
| Ⅱ | 8/50 (16.0) | 18/126 (14.3) |  |
| Ⅲ | 40/50 (80.0) | 103/126 (81.7) |  |
| ISS stage |  |  | 0.980 |
| Ⅰ | 12/50 (24.0) | 32/127 (25.2) |  |
| Ⅱ | 18/50 (36.0) | 44/127 (34.6) |  |
| Ⅲ | 20/50 (40.0) | 51/127 (40.2) |  |
| M component |  |  | 0.160 |
| IgG | 23/50 (46.0) | 67/127 (52.8) |  |
| IgA | 19/50 (38.0) | 27/127 (21.3) |  |
| IgD | 1/50 (2.0) | 9/127 (7.1) |  |
| Light chain | 7/50 (14.0) | 23/127 (18.1) |  |
| Nonsecretory | 0/50 (0.0) | 1/127 (0.8) |  |
| Hemoglobin (g/L) | 90 (44-133) | 97 (48-146) | 0.126 |
| Calcium (mmol/L) | 2.42 (1.80-3.43) | 2.38 (1.65-4.29) | 0.388 |
| Lactate dehydrogenase (U/L) | 151 (94-447) | 154 (67-732) | 0.714 |
| Serum creatinine (μmol/L) | 78.3 (41.1-745.0) | 74.8 (30.0-881.0) | 0.812 |
| Albumin (g/L) | 35.5 (17.0-46.0) | 34.5 (18.0-55.0) | 0.209 |
| β2-microglobulin (mg/L) | 3.72 (0.76-20.60) | 3.34 (0.63-28.66) | 0.565 |
| Data are presented as n (%) or median (range)  * means p<0.05 | | | |
